# Supplementary material for: Analysis of the Volatile Components in Selaginella doederleinii by Headspace Solid Phase Microextraction-Gas Chromatography-Mass Spectrometry
Source: Molecules. 2019 Dec 27;25(1):115. doi: 10.3390/molecules25010115 (PMC6982779; doi:10.3390/molecules25010115)
Supplement: Supplementary file 1 [file molecules-25-00115-s001.pdf]

1. Mass spectrometric analysis of four volatile oils

Based on GC-MS analysis, terpene was the main component of *Selaginella doederleinii*, among which β-caryophyllene and β-asarone had the wide range of biological activity[1-5]. In addition, fitone and anethol were the highest percentage compounds of in SD Guizhou and SD Zhengjiang, respectively. Therefore, β-caryophyllene, anethol, β-asarone and fitone were selected to analyze fragment information in the mass spectrum and compared with those of MS interpreter in NIST software.

Figure S1-A1 and Figure S1-A2 showed the mass spectrum of peak 19 and anethol in MS interpreter, respectively. The retention time of peak 19 was 17.727 min. It could be seen that its fragment ion peaks were m/z 117, 77 and 55, respectively. This might be a methoxyl group and aromatic hydrocarbons formed by the cleavage of an ether bond on target molecule. It was speculated that there were an aromatic structure in the compound by the fragment ion peaks of $[C_4H_7]^+$ and $[C_6H_5]^+$ at m/z 55 and 77, respectively.

Figure S1-B1 was the mass spectrum of peak 31, and Figure S1-B2 was the mass spectrum of β-caryophyllene in MS interpreter. The retention time of peak 31 was 21.379 min, and a series of prominent ions at m/z 189, 161, 133, 93, 69 and 41 were observed. It was worth noting that the characteristic fragment ion peaks of hydrocarbon compounds such as $[M-CH_3]^+$, $[M-C_3H_7]^+$, $[C_5H_9]^+$ and $[C_3H_4]^+$ at m/z 189, 161, 69 and 41, respectively, were detected.

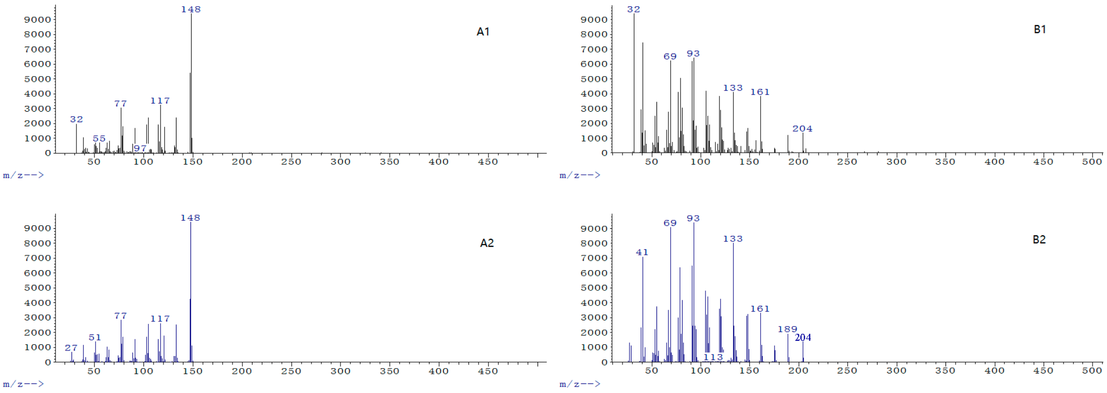

Figure S1. Mass spectrum of peak 19 (A1) and anethol (A2); Mass spectrum of peak 31 (B1) and β-caryophyllene (B2).

Figure S2-A1 and Figure S2-A2 showed the mass spectrum of peak 49 and β-asarone in MS interpreter, respectively. The retention time of peak 49 was 28.575 min, and the fragment ions were exhibited at m/z 193, 165, 137, 91, 69 and 41. It could be inferred that the target molecule lost a series of CO and methyl continuously and produced the fragment ions of $[M-CH_3]^+$, $[M-CH_3-CO]^+$ and $[M-CH_3-CO-CO]^+$ at m/z 193, 165 and 137, respectively. The fragment ions of $[C_7H_7]^+$ at m/z 91 and $[C_5H_9]^+$ at m/z 69 showed that it was an aromatic compound. Moreover, the characteristic fragment ion of $[CH_3O]^+$ at m/z 41 indicated that the compound contained $-OCH_3$.

Figure S2-B1 was the mass spectrum of peak 58, and Figure S2-B2 was the mass spectrum of fitone in MS interpreter. The retention time of peak 58 was 37.812 min, and a series of prominent ions at m/z 253, 124, 95, 71, 55 and 43 were observed. It was worth noting that the fragment ion peak of $[C_2H_3O]^+$ at m/z 43 was high-abundant signal and indicated that the compound contained a acetyl group that produced the characteristic cleavage of ketone. In addition, the characteristic fragment ion peaks of hydrocarbon compounds such as $[M-CH_3]^+$, $[M-C_2H_3O-C_7H_{16}]^+$, $[C_7H_{11}]^+$ $[C_5H_{11}]^+$ and $[C_4H_7]^+$ at m/z 253, 124, 95, 71 and 55, respectively, were also detected, which indicated there was an aromatic structure in the compound.

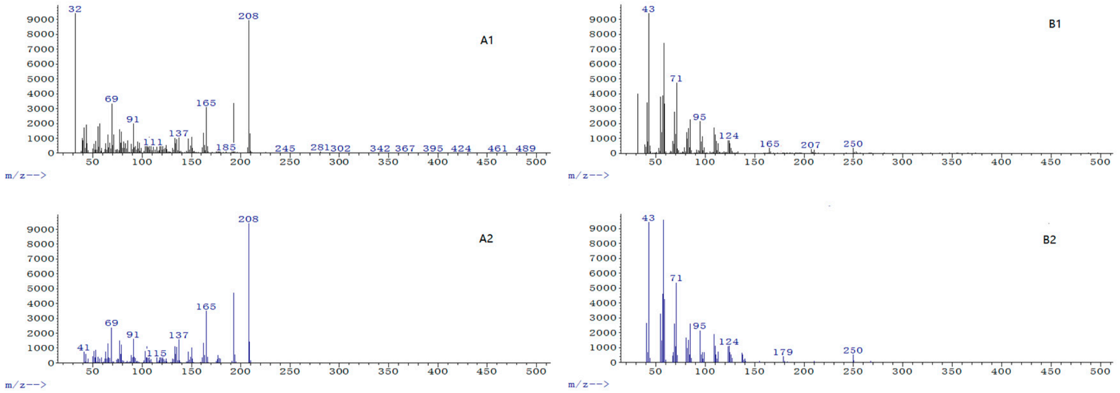

Figure S2. Mass spectrum of peak 49 (A1) and β-asarone (A2); Mass spectrum of peak 58 (B1) and fitone (B2).

2. Sample collection geodata informations

A total of 11 batches of Zhejiang SD (batch number ZJ01, ZJ02, ZJ03, ZJ04, ZJ05, ZJ06, ZJ07, ZJ08, ZJ09, ZJ10 and ZJ11 and 13 batches of Guizhou SD (batch number GZ01, GZ02, GZ03, GZ04, GZ05, GZ06, GZ07, GZ08, GZ09, GZ10, GZ11, GZ12 and GZ13) were purchased from Hangzhou Chinese Herbal Medicine Trading Center (Zhejiang Province) and Guiyang Chinese Herbal Medicine Market (Guizhou Province) (See Table S1). These samples were authenticated by Zhang Yujin (an associate professor from Zunyi Medical University) and deposited in the chemical laboratory.

Table S1. Sample collection geodata informations

Sample	Date of Collection	Origin	Collection Site
ZJ01	07-2018	Hang Zhou, Zhejiang, China	Qiandao Lake
ZJ02	07-2018	Hang Zhou, Zhejiang, China	Qiandao Lake
ZJ03	07-2018	Hang Zhou, Zhejiang, China	Qiandao Lake
ZJ04	07-2018	Hang Zhou, Zhejiang, China	Niangniang shan
ZJ05	07-2018	Hang Zhou, Zhejiang, China	Niangniang shan
ZJ06	07-2018	Hang Zhou, Zhejiang, China	Niangniang shan
ZJ07	07-2018	Hang Zhou, Zhejiang, China	Lion Rock
ZJ08	07-2018	Hang Zhou, Zhejiang, China	Lion Rock
ZJ09	07-2018	Hang Zhou, Zhejiang, China	Lion Rock
ZJ10	07-2018	Hang Zhou, Zhejiang, China	Lingshan
ZJ11	07-2018	Hang Zhou, Zhejiang, China	Lingshan
GZ01	06-2018	Guiyang, Guizhou, China	Qingshan
GZ02	06-2018	Guiyang, Guizhou, China	Qingshan
GZ03	06-2018	Guiyang, Guizhou, China	Qingshan
GZ04	06-2018	Guiyang, Guizhou, China	Baiyan Mountain
GZ05	06-2018	Guiyang, Guizhou, China	Baiyan Mountain
GZ06	06-2018	Guiyang, Guizhou, China	Baiyan Mountain
GZ07	06-2018	Guiyang, Guizhou, China	Baihua Reservoir
GZ08	06-2018	Guiyang, Guizhou, China	Baihua Reservoir
GZ09	06-2018	Guiyang, Guizhou, China	Baihua Reservoir
GZ10	06-2018	Guiyang, Guizhou, China	Jiutou Mountain

GZ11	06-2018	Guiyang, Guizhou, China	Jiutou Mountain
GZ12	06-2018	Guiyang, Guizhou, China	Jiutou Mountain
GZ13	06-2018	Guiyang, Guizhou, China	Jiutou Mountain
